# An exploratory psychometric network analysis of loneliness scales in a sample of older adults

**DOI:** 10.1007/s12144-023-04697-9

**Published:** 2023-05-13

**Authors:** Alexandra Thompson, Thomas V. Pollet

**Affiliations:** Department of Psychology, Northumbria University, Newcastle Upon Tyne, UK

**Keywords:** Loneliness, Friendships, Older Adults, Psychological Well-being

## Abstract

To examine the relationships within and between commonly used measures of loneliness to determine the suitability of the measures in older adults. Further, to determine whether certain items in these measures are more psychometrically robust in terms of capturing different types of loneliness across this population. Data were obtained from 350 older adults via completion of an online survey. Four measures of loneliness were completed. These were the University of California Los Angeles Loneliness Scale 4 (Version 3), the de Jong Gierveld Loneliness Scale, the Social and Emotional Loneliness Scale for Adults (Short Version) and a direct measure of loneliness. Analysis via a regularized partial correlation network and via clique percolation revealed that only the SELSA-S encompassed loneliness relating to deficits in social, family and romantic relationships. The remaining measures tapped mostly into social loneliness alone. The direct measure of loneliness had the strongest connection to the UCLA item-4 and the de Jong Gierveld item-1 exhibited the strongest bridge centrality, being a member of the most clusters. The results indicate that should researchers be interested in assessing loneliness resulting from specific relationships, then the SELSA-S would be the most suitable measure. Whereas the other measures are suitable for assessing loneliness more generally. The results further suggest that the de Jong Gierveld item-1 may be a more suitable direct measure of loneliness than that currently employed as it taps into a greater number of relationships.

## Introduction

Loneliness has been defined as an unpleasant or distressing experience resulting from a perceived qualitative or quantitative deficiency in one’s social relationships (Peplau & Perlman, 1982; Russell et al., 1980). Transient experiences of loneliness are believed to be adaptive in that they provide motivation to form and maintain social connections in order to promote the survival of genes (Cacioppo & Hawkley, 2009; Cacioppo et al., 2006; Hawkley & Cacioppo, 2010). However, sustained loneliness has been repeatedly linked to negative psychological and physiological health outcomes. In terms of psychological outcomes, these include but are not limited to anxiety and depression (Age Uk South Lakeland, 2018; Barg et al., 2006; Cacioppo et al., 2010), suicidality (Stravynski & Boyer, 2001; Van Orden et al., 2010), maladaptive stress responses (Adam et al., 2006; Steptoe et al., 2004), cognitive decline and Alzheimer’s Disease (Boss et al., 2015; Donovan et al., 2017; Wilson et al., 2007). In terms of physiological outcomes, this includes cardiovascular disease (Momtaz et al., 2012; Valtorta et al., 2016, 2018), malnutrition (Ramic et al., 2011), sleep quality (Yu et al., 2018), functional decline (Perissinotto et al., 2012) as well as increased risk of mortality (Holt-Lunstad et al., 2010, 2015).

Although loneliness impacts individuals of all ages (Barreto et al., 2020; Pyle and Evans, 2018; Schultz and Moore, 1988), it is clear that loneliness is prevalent in older adults as well as younger age groups. For example, in excess of 1 million UK residents over the age of 50 report that they are chronically lonely (Abrahams, 2018). This figure is expected to increase to 2 million by 2025 (Abrahams, 2018). Additionally, the pooled prevalence of loneliness has been estimated to be at around 29% for adults aged 65 and over for 29 high income countries (Chawla et al., 2021). This is cause for concern given the vast array of negative outcomes associated with loneliness and the physiological vulnerability that many older adults experience (MacNee et al., 2014). As well as directly impacting the lived experiences of these older adults, there are implications in terms of increased health and social care service utilisation and associated economic costs. This potential for harm, coupled with the ageing population worldwide (UN DESA’s Population Division, 2019), suggests that it is important that loneliness in this age group is understood in order to develop strategies to counteract these negative impacts.

### The conceptualisation of loneliness

When discussing loneliness and its negative impacts, it is important to note that although social isolation and loneliness are conceptually similar, they are separate constructs. Social isolation refers to an objectively measured shortfall in an individual’s social relationships such as few network ties and low frequency of contact with those ties (Holt-Lunstad et al., 2015). Loneliness is a perceived deficit between actual and desired quality or quantity of relationships (Peplau & Perlman, 1982). As such, individuals who are quantifiably socially isolated may not experience loneliness whereas those with many social connections can experience loneliness (Donovan & Blazer, 2020).

The construct of loneliness has been conceptualised as both a unidimensional as well as a multidimensional construct. A seminal multidimensional model of loneliness was suggested by Weiss (1973). This model proposed that loneliness consisted of two dimensions: social and emotional loneliness. He suggested that loneliness in each of these dimensions resulted from a deficit in qualitatively different relationships: affiliations and attachments. Affiliations were suggested to be relationships such as friendships and work acquaintances. In contrast, attachments were suggested to be close, intimate relationships such as romantic relationships or parent–child bonds (Weiss, 1973). Later work in developing measurement instruments to assess loneliness has resulted in the creation of various constructs capturing differing facets of this experience which are discussed below.

### The measurement of loneliness

Some of the most commonly employed measures of loneliness are the 11-item de Jong Gierveld Scale (dJG) (de Jong-Gierveld & Kamphuis, 1985), the University of California, Los Angeles, Loneliness Scale-Version 3 (UCLA-3) (Russell, 1996), and the shortened version of the Social and Emotional Loneliness Scale (SELSA-S) (DiTommaso et al., 2004). These measures have consistently been found to be reliable in a variety of populations (e.g., Adamczyk & DiTommaso, 2014; Buz et al., 2014; Russell, 1996) but differ in terms of dimensionality, length and response categories.

#### The 11-item de Jong Gierveld loneliness scale

The 11-item de Jong Gierveld Scale (dJG) (de Jong-Gierveld & Kamphuis, 1985), aims to capture the multidimensional nature of loneliness as proposed by Weiss (1973). However, there has been some debate with regards to the factorial structure of the dJG scale. Support for a unidimensional structure with no grounds for bi-dimensionality has been evidenced (Buz & Perez-Arechaederra, 2014; Buz et al., 2014; de Jong-Gierveld & Kamphuis, 1985). However, models proposing a two-factor structure based on item wording or qualitative differences have also been supported (e.g., Buz et al., 2014; de Jong Gierveld et al., 2006; de Jong-Gierveld & van Tilburg, 1999; Penning et al, 2014; van Baarsen et al., 2001).

#### The social and emotional loneliness scale for adults

The Social and Emotional Loneliness Scale for Adults (SELSA) was also developed based on Weiss’ (1973) typology (DiTommaso et al., 2004). It is intended to capture both social and emotional loneliness and further subdivides emotional loneliness into romantic and family elements (DiTommaso & Spinner, 1993). The original scale included 37 items, it was shortened to a 15 item scale, dubbed the SELSA-S (DiTommaso et al., 2004). It has been demonstrated to have a three-factor structure in line with the three separate subscales. This three factor structure has been further supported in Turkish, Persian and Polish validations of the scale (Adamczyk & DiTommaso, 2014; Cecen, 2007; Jowkar & Salimi, 2012).

#### The University of California Los Angeles (UCLA) loneliness scale

The UCLA scale focuses on measuring loneliness in relation to various social relationships rather than distinguishing specifically between social and emotional loneliness. As such, this measure was intended to capture a single global dimension of loneliness (Russell et al., 1978). The scale has undergone a variety of revisions to improve clarity and avoid acquiescence bias resulting in the oft employed Version 3 of the scale (Russell, 1996; Russell et al., 1980).

From its inception there has been a debate as to whether loneliness as measured by this scale has a unidimensional structure or rather consists of multiple dimensions. For example, models suggesting a single global bipolar factor along with two method factors accounting for item-wording have been supported in a variety of populations (Durak & Senol-Durak, 2010; Eglit et al., 2018; Russell, 1996). Alternative three-factor models have been proposed such as being comprised of factors representing Isolation, Relational Connectedness and Trait Loneliness (Boffo et al., 2012) as well as by Isolation (relating to feelings of aloneness and rejection), Related Connectedness (concerning attachments to other individual), and Collective Connectedness (addressing group affiliation) (Hudiyana et al., 2021; Shevlin et al, 2015). Despite the mixed findings surrounding the underlying structure of the UCLA-3, this measure is typically treated as measuring a unidimensional construct of loneliness as originally intended by the scale authors (e.g., DiTommaso et al., 2004).

### Scale choice

As has been discussed, a variety of conceptualisations and measurement instruments to assess loneliness exist. This multitude of measurement options with the ability to capture differing aspects of loneliness makes it challenging for practitioners and researchers to determine which measure is most suitable to use, especially in a particular population, such as in older adults.

Moreover, as described above, there is some ongoing debate regarding the underlying factorial structure of the majority of these scales. It is therefore unclear which types of loneliness each of these scales tap into or whether, in contrast, we can treat loneliness as a unitary construct. However, if loneliness is indeed multidimensional, knowledge of which type of loneliness can be assessed by which scale is important as it may have implications for the choice of measurement tool and the development of interventions. For example, loneliness resulting from a deficit in an emotional attachment is unlikely to be improved by increasing social interactions, rather an intervention focusing on strengthening. Selecting one scale over another could mean that a pertinent aspect of loneliness for the population or research question of interest is missed.

It is also not known whether one scale has an advantage over another in measuring loneliness in that they incorporate key items for assessing specific dimensions. Further, some items may overlap in that they are worded similarly (e.g., dJG-8: *There are enough people I feel close to* and UCLA-10: *How often do you feel close to people?*) and others may be more distinct. This overlap could further make it difficult for practitioners and researchers to decide on a measurement tool. Clarity in terms of key items and assessable domains could enable the most effective tool to be selected.

### A network approach

A novel way to approach this is to estimate the relationships between all items, as a network (e.g., Manson et al., 2020); Armour et al., 2017; Christensen et al., 2018; VanRooijen, 2017). This allows the reciprocal relationships between the items to be estimated and visualised. Estimation of a partial correlation network allows connections (edges) between nodes (in this case scale items) to be represented as correlation coefficients after controlling for all other edges in the network. Thereby enabling the strength of these connections to be intuitively understood.

Further, this visualisation can lead to the detection of groupings within the network indicative of the presence of latent variables (Epskamp & Fried, 2018). Therefore, allowing the underlying factor structure of these scales to be explored. Moreover, this visualisation enables us to determine which scales overlap and therefore which types of loneliness each scale is able to assess. These groupings are often referred to as communities or clusters. Within the present paper these groupings will be referred to as clusters throughout.

The reciprocal nature of the connections within the network also enables us to better understand the interrelationships between loneliness ‘symptoms’. It may be that certain dimensions of loneliness are more strongly related to others. For example, it could be that items related to romantic loneliness, as captured with the SELSA, are more strongly connected to an item from the UCLA like: ‘How often do you feel that you lack companionship?’ than items relating to family loneliness. This would have important practical implications for which social relationships to target. Observing scale items (symptoms) this way has been beneficial in the field of psychopathology such as with PTSD (e.g., Armour et al., 2017), however this has not yet been applied to loneliness.

An additional benefit over traditional factor analysis techniques is that items can be assessed to determine their centrality within the network. Whereas more commonly used methods aim to reduce cross-loading between factors, assessing this centrality between factors gives an indication of a node’s connectivity between factors. As well, traditional factor analytic approaches tend to rely on the conception of latent variables and assumes causality rather than taking into account potential reciprocal or cyclical relationships which are likely present within psychological symptoms. Despite these benefits, such analyses appear not yet to have been applied to such measures of loneliness.

### The present study

As outlined above, the use of a network approach to examine the interrelationships within and between commonly utilised loneliness measures is warranted. To this end, the present study aimed to extend previous findings in the following key ways. First, we estimated a network containing all items from the four loneliness measures via a regularised partial correlation model. This allowed us to determine which items had the strongest connections; of particular interest were those relating to the direct measure of loneliness. This also allowed us to examine the underlying structure of the measures by visually assessing clustering of items. Clique percolation was used to further identify clusters within the network and to identify items with strong connections to more than one cluster. Bridge centrality statistics were also estimated to determine which items had the most connectivity between these clusters.

In terms of factorial structure, we expected to find a two factor structure for the DJG scale in line with previous findings. For the SELSA-S we expected to uncover a three factor structure in line with the three proposed subscales. Given the mixed findings for the UCLA scale in relation to factorial structure, we expected to uncover a three factor structure. However, the items belonging to each factor remain exploratory in nature. This is because a variety of differing three factor solutions have been recovered (e.g., Boffo et al., 2012; Sancho et al., 2020; Shevlin et al., 2015). We expected to observe some overlap between the types of loneliness that each scale is able to assess given scale item similarity and the theoretical basis of loneliness each scale was designed on. As such we tentatively expected that there may be some overlap between the social loneliness aspects of all of the scales possibly forming one cluster and overlap between the emotional loneliness aspects of the DJG scale and the SELSA-S potentially forming another cluster.

## Method

### Design

This was a cross-sectional network analysis of scale item scores across four measures of loneliness in older adults.

### Sample

A target sample size of 400 was pre-registered, this was primarily determined by cost and a heuristic that the sample sizes would be sufficient for structural equation modelling (SEM) (e.g., Barrett, 2007). The pre-registered sample was deemed to be sufficient for SEM (for further details see the pre-registration on the Open Science Framework (OSF) page). A total of 350 residents of the United Kingdom aged 65 or over responded to an online questionnaire. This was advertised via the recruitment platform Prolific (Palan & Schitter, 2018), via social media posts and via word of mouth. Recruitment was terminated once further responses ceased for three consecutive days. Those that completed the questionnaire via Prolific were paid £2 upon completion (n = 290). To be eligible to take part in the study participants were required to not have had a current clinical diagnosis of depression or anxiety. The requirement for a lack of diagnosis is due to ethical concerns of these participants completing the measures in our survey, as well as potential confounding. The initial sample consisted of 350 respondents (138 male, 211 female and one participant did not specify).

### Measures

Four measures of loneliness were employed within this study. These were as follows:

#### The 11-item de Jong Gierveld loneliness scale

The 11-item de Jong Gierveld Scale (dJG) (de Jong-Gierveld & Kamphuis, 1985). This scale includes two sub-scales. An emotional loneliness sub-scale consisting of 6 negatively worded items and a social loneliness subscale consisting of 5 positively worded items. Respondents are asked to indicate how much each statement applies to their current situation (e.g., I miss having a really close friend). Response options were adapted from the original version (yes!, yes, more or less, no, no!) to improve their interpretation. The options were ‘All of the time’, ‘Often’, ‘some of the time’, ‘Rarely’ and ‘None of the time’. Responses in the original dJG are dichotomously scored by summing the positive and neutral responses for the emotional loneliness items and by summing the negative and neutral responses for the social loneliness items. The scale can also be utilised as a global unidimensional measure of loneliness by summing the scores for both sub-scales. Greater scores indicate greater levels of loneliness. In the present study, based on previous critiques (e.g., van Baarsen et al., 2001), and as implemented in previous studies (e.g., Penning et al., 2014; van Tilburg et al., 2004), we instead reverse-scored non-lonely items and did not dichotomize scores based on valence, but rather use the entire range. Internal reliability has previously been found to be good for the global scale as well as for both subscales (All Cronbach’s $\alpha$ > 0.80) (Grygiel et al., 2019). Reliability for the global scale in the present study was found to be excellent (Cronbach’s $\alpha$ = 0.92). For the subscales, the internal reliability was found to be good: emotional loneliness (Cronbach’s $\alpha$ = 0.89) and social loneliness (Cronbach’s $\alpha$ = 0.88).

#### The University of California Los Angeles loneliness scale version 3

The University of California, Los Angeles, Loneliness Scale (Version 3) (UCLA-3) (Russell, 1996). The UCLA-3 is a 20-item self-report scale designed to measure an individual’s subjective feelings of loneliness. Participants are asked how often each statement is descriptive of them (e.g., How often do you feel that you lack companionship?). Items are on a scale ranging from 1 (Never) to 4 (Often) and nine items are reverse scored (non-lonely). As the scale is intended to capture a unidimensional construct of loneliness, all items are summed to give a single loneliness score; a higher score represents greater levels of loneliness. Internal reliability has previously been found to range from good (Cronbach’s $\alpha$ = 0.89) to excellent (Cronbach’s $\alpha$ = 0.91) (Russell, 1996). Internal reliability for this scale in the present study was found to be excellent (Cronbach’s $\alpha$ = 0.94). Previously reported reliability for the commonly reported three subscales has been found to be: Isolation—Cronbach’s $\alpha$ = 0.85, Related Connectedness—Cronbach’s $\alpha$ = 0.74, Collective Connectedness—Cronbach’s $\alpha$ = 0.70. For the commonly reported three subscales in the present study, reliability was as follows: Isolation—Cronbach’s $\alpha$ = 0.94, Related Connectedness—Cronbach’s $\alpha$ = 0.94, Collective Connectedness—Cronbach’s $\alpha$ = 0.94) (Shevlin et al., 2015).

#### The short social and emotional loneliness scale

The shortened version of the Social and Emotional Loneliness Scale (SELSA-S) (DiTommaso et al., 2004) was developed in order for the resulting scale to be similar in length to other commonly used measures of loneliness and to allow for efficient measurement in clinical settings (DiTommaso et al., 2004). The SELSA-S is a 15-item multidimensional measure of loneliness designed to measure both social and emotional loneliness. The family division of the emotional loneliness subscale is believed to assess loneliness resulting from attachment relationships with family, whereas the romantic subscale is thought to concern loneliness relating to romantic relationships. The SELSA-S consists of three subscales: social, emotional family and emotional romantic loneliness. Each subscale consists of 5 items. Items are on a scale ranging from 1 (Strongly Disagree) to 7 (Strongly Agree) and three items are reverse scored for each subscale. Higher scores represent higher levels of loneliness. A total emotional loneliness score can be obtained by summing the family and romantic subscales. For the subscales, reliability has been found to range from good to excellent: family subscale (Cronbach’s $\alpha$ = 0.89), romantic subscale (Cronbach’s $\alpha$ = 0.87) and the social subscale (Cronbach’s $\alpha$ = 0.90) (DiTommaso et al. (2004)). Internal reliability for the total emotional loneliness scale in the present study was found to be good (Cronbach’s $\alpha$ = 0.86) as was the reliability for the family subscale (Cronbach’s $\alpha$ = 0.89) and the romantic subscale (Cronbach’s $\alpha$ = 0.84). Internal reliability for the social emotional loneliness subscale was also found to be good (Cronbach’s $\alpha$ = 0.81).

#### Other measures

A direct measure of loneliness, ‘Do you feel lonely?’, which has been used to assess loneliness in a variety of studies previously (e.g., Holmen & Furukawa, 2002; Nicolaisen & Thorsen, 2014; Routasalo et al., 2006). This had four response categories: Often, Sometimes, Seldom and Never. This measure was reverse scored. This question has been reported to have good predictive and face validity (Routasalo et al., 2006). Other measures were collected but are not reported here. These include the short version of the Depression Anxiety and Stress Scale (DASS-21) (Lovibond & Lovibond, 1995), the Satisfaction with Life Scale (SWLS) (Diener et al., 1985), and respondent’s ideal and actual number of close friends. These variables are reported on elsewhere (Thompson et al., 2022). The design of the study, questionnaire and all variables are available from the OSF.

### Procedure

Ethical approval was granted by the local ethics committee (NAME REMOVED FOR REVIEW—Reference 16,881). After informed consent was obtained, participants completed all measures via an online questionnaire hosted by (“Qualtrics Survey Software,” 2020) available at https://www.qualtrics.com. The demographic measures were completed first followed by each of the loneliness scales (including the direct measure). The order in which these scales were presented were randomised. This was followed by completion of the SWLS, DASS-21 and the participant’s number of actual and ideal close friends. The whole questionnaire took no longer than 20 min to complete.

### Analytical approach

All analyses were performed using R version 3.6.1 (R Core Team, 2017). Two cases that contained missing data for some of the loneliness scale items were removed. This resulted in a final sample of 348 participants. Data and code for this manuscript are available on the OSF.

#### Network estimation

In order to ascertain the edge weights between all items and to determine underlying factor structure, a regularized partial correlation network containing all of the items from each of the four loneliness measures was estimated via the ‘bootnet’ package (Epskamp et al., 2018). Here, items were colour coded based on the theoretical loneliness scale they belong to in order to visualise clustering. The regularization technique employed was the Graphical LASSO (Least Absolute Shrinkage and Selection Operator) with EBIC (Extended Bayesian Information Criterion) tuning parameter, in line with recommendations of Epskamp and colleagues (Epskamp & Fried, 2018; Epskamp et al., 2018). The tuning parameter automatically selects the ‘best’ network model by optimising the fit of the network to the data by minimising the EBIC. The EBIC hyperparameter determines how much the EBIC prefers simpler models over more complex models. In this instance, the hyperparameter was set to 0.5 which dictates a stronger preference for models which retain the fewest edges. As noted by Epskamp (2018) the choice of hyperparameter value depends on the goals of study in question. This particular hyperparameter was chosen based on the expected dense structure of the current model. Here, spurious edges shrink to exactly zero and drop out of the model resulting in a sparse model. Thus, increasing interpretability of the model. Unsurprisingly, as some of the scales only have 4 response categories, the assumption of multivariate normality was violated. In order to handle this, the ‘corMethod’ argument is set to ‘corAuto’ in bootnet, which leads to the detection of ordinal data and the computation of polychoric correlations, which are then carried forward in the network estimation algorithm. This process overcomes the need to transform the data (Epskamp & Fried, 2018).

Once the edge weights had been estimated they were bootstrapped via a non-parametric method which constructs 95% confidence intervals around the estimated edge weights. This provides an indication of the accuracy of the edge weight estimates.

#### Cluster identification

Many traditionally used clustering methods force nodes (in this case, items) to be part of one cluster only such as the walktrap algorithm method (Pons & Latapy, 2005). However, it is not uncommon for items to cross-load, therefore it might be unrealistic to assign items to only a single cluster. Therefore, a weighted clique percolation plot was generated using the ‘CliquePercolation’ package (Lange, 2019). This allows to further elucidate the underlying factor structure and to determine which nodes may belong to more than one cluster. Here, the clusters are not explicitly defined based on the theoretical loneliness measure the items belong to and are instead determined via an algorithm. The algorithm implemented within this package requires that researchers optimise *i* (strength of the partial correlation between nodes) and *k* (minimum clique or cluster size) for the network in question. In line with recommendations, this optimisation is based on the ratio threshold exceeding a value of 2 (Fried, 2019).

#### Bridge centrality estimation via bridge expected influence

**Fig. 1:**
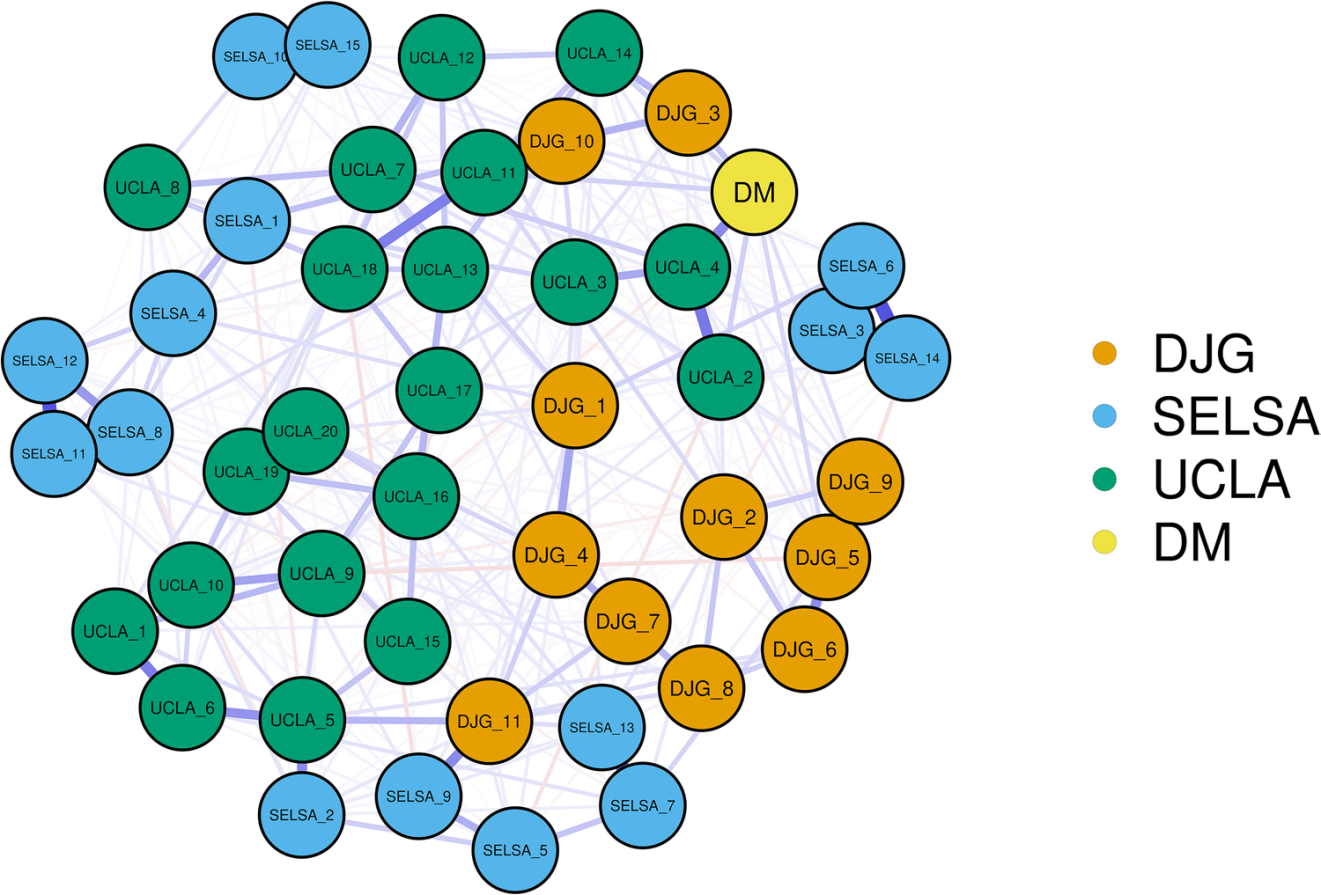
Regularised partial correlation network containing items from all loneliness measures. dJG = de Jong Gierveld scale, UCLA = University of California Los Angeles scale, SELSA = Social and Emotional Loneliness Scale for Adults, DM = Direct measure of loneliness

Network centrality indices are commonly used to determine a node’s (or in this case an item’s) relative importance within the network (e.g., Rodrigues, 2019). Of particular interest, were the nodes which displayed the strongest connectivity with separate measures of loneliness. Here, the clusters were explicitly defined based on the theoretical loneliness scales that the items belong to (See Fig. 1). Items which displayed connectivity between the separate scales (clusters) were then identified via a particular bridge centrality estimation, bridge expected influence. Bridge expected influence is a centrality measure which, in this instance, estimates a node’s sum connectivity with separate measures of loneliness (Jones et al., 2021); these nodes act as a bridge between separate loneliness measures. Although the suitability of centrality estimation for psychological networks has been questioned (Bringmann et al., 2019), expected influence and strength have been proposed to be more appropriate in comparison to betweenness and closeness. Additionally, bridge expected influence and bridge strength were devised for the purpose of identifying key psychometric items which act as bridges between theoretically determined psychological communities (Jones et al., 2021).

The stability of this centrality measure was assessed via a case drop bootstrap via the ‘bootnet’ package. The results of this bootstrap can be summarised via a correlation stability coefficient. This coefficient indicates the proportion of cases that can be dropped to retain with 95% confidence a correlation of at least 0.7 with the original coefficient. Ideally, the value of this coefficient should be above 0.5, however, a value above 0.25 is deemed acceptable (Epskamp & Fried, 2018).

There are multiple other network centrality metrics which could be derived for a psychometric network analysis, these are reported in full on the OSF.

## Results

Sample characteristics and descriptive statistics are presented in Table 1.

**Table 1:** Sample characteristics (*n*=348)

Characteristics	Statistics (Mean (SD) or *n* (%))
Age	69.44 (4.35)
Gender	
Female	209 (60)
Male	137 (39)
Undisclosed	2 (<1)
Marital Status	
Single	19 (6)
In a relationship	28 (8)
Married/ Civil Partnership	215 (62)
Separated	5 (1)
Divorced	51 (16)
Widowed	29 (8)
Undisclosed	1 (<1)
Education	
Primary school	2 (<1)
Some secondary school	30 (9)
GCSE’s or equivalent	67 (20)
A level or equivalent	70 (20)
Undergraduate degree	118 (34)
Postgraduate degree	59 (17)
Undisclosed	2 (<1)
UCLA	40.17 (11.76)
DJG Total	2.22 (1.94)
DJG Emotional	2.19 (2.14)
DJG Social	2.22 (1.94)
SELSA Social	15.3 (6.44)
SELSA Emotional	28.82 (12.95)
SELSA Family	11.97 (6.95)
SELSA Romantic	16.86 (6.68)

### Regularised partial correlation network estimation

For scale item descriptions please see Appendix A. Visual inspection of the initial regularised partial correlation network revealed a mostly unitary structure for the UCLA-3 (Fig. 1). For the dJG, two groupings emerged. One grouping consisted of a dyad representing the two emotional loneliness nodes examining emptiness and rejection (dJG-3 & dJG-10). The second grouping consisted of the remaining dJG nodes with no further distinction between social and emotional loneliness. For the SELSA-S, there appear to be four groupings. All social loneliness nodes comprised one group. All family emotional loneliness nodes comprised the second grouping. For romantic emotional loneliness, a further distinction was made between the positively worded and negatively worded items. The plot is displayed in Fig. 1. Within this plot the items have been colour coded based on the respective scales they theoretically belong to.

Of note, the direct measure of loneliness (*Do you feel lonely?*) had the strongest partial correlation with the UCLA-4 item (How often do you feel alone?) (*r* = 0.243, 95%CI [0.141, 0.338]; raw correlation *rs* = 0.73). The next four strongest connections were with: the dJG-3 item (*I experience a general sense of emptiness*) (*r* = 0.128, 95%CI [0.034, 0.221]), the UCLA-2 item (*How often do you feel that you lack companionship?*) (*r* = 0.123, 95%CI [0.020, 0.230]), the dJG-5 item (*I miss the pleasure of the company of others*) (*r* = 0.093, 95%CI [0.007, 0.3179]), the UCLA-11 item (*How often do you feel left out?*) (*r* = 0.095, 95%CI [0.014, 0.178]).

Some overlap between the bootstrapped confidence intervals for the edge weights can be seen here, implying that the order of the edge weights should be interpreted with caution. However, the overlap is much less marked for the edge between the direct measure and the UCLA-4 item, suggesting some confidence that this is the most strongly connected item to the direct measure.

### Clique percolation cluster identification

**Fig. 2:**
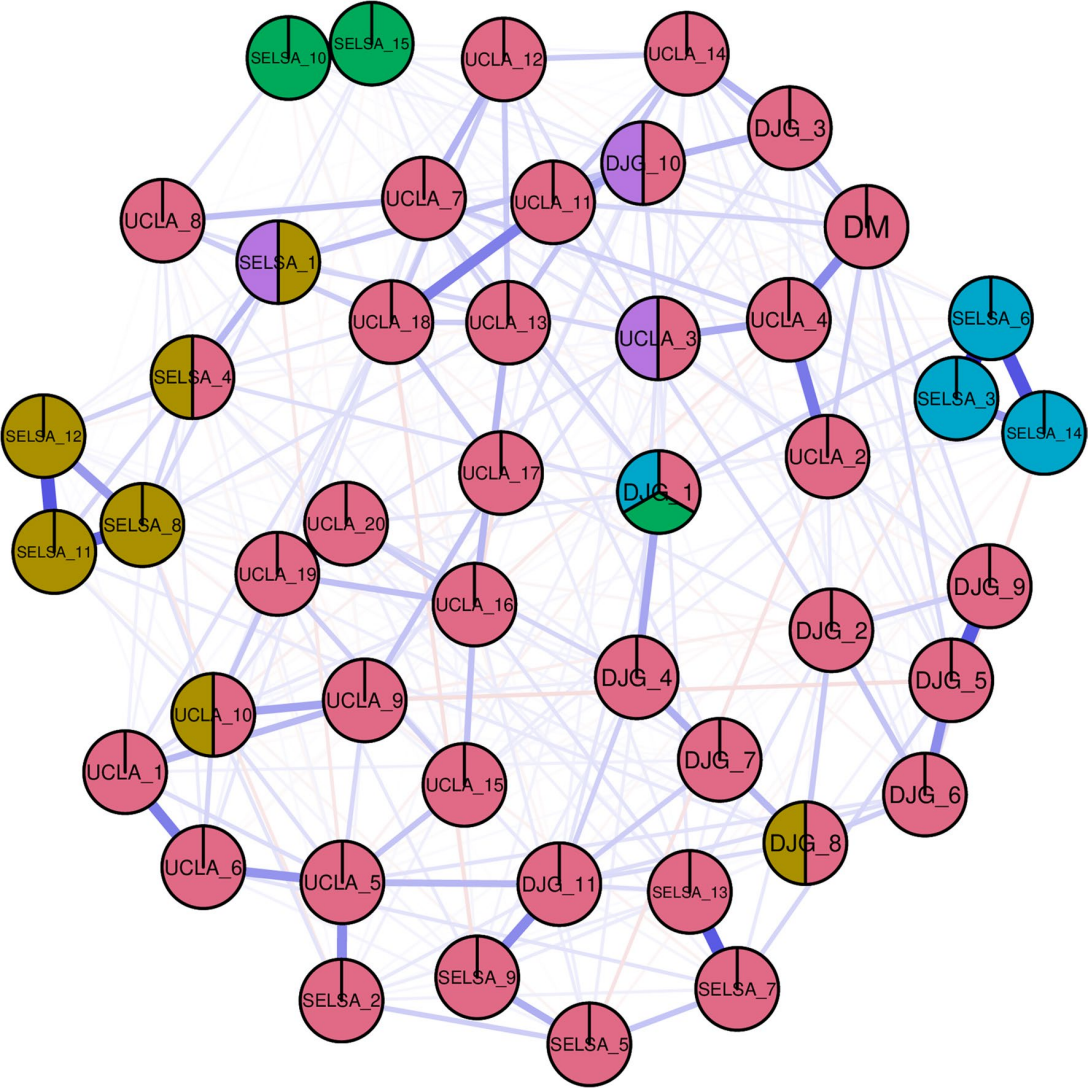
Clique percolation network containing items from all loneliness measures. dJG = de Jong Gierveld scale, UCLA = University of California Los Angeles scale, SELSA = Social and Emotional Loneliness Scale for Adults, DM = Direct measure of loneliness

Inspection of the Clique Percolation plot identified five clusters as outlined below. This plot is displayed in Fig. 2:

The first cluster consisted of the SELSA-S romantic loneliness nodes that were positively worded (non-lonely e.g., *I have a romantic partner with whom I share my most intimate thoughts and feelings*) which comprised one cluster as found in the previous network estimation but with the addition of the dJG-3 node (*‘I experience a general sense of emptiness’*).

The SELSA-S negatively worded romantic loneliness nodes comprised a second cluster as in the previous estimation. However, this cluster also included dJG-1.

The SELSA-S family emotional loneliness nodes made up the majority of a third cluster. This cluster also included UCLA-10 (*‘How often do you feel close to people?’*) and dJG-8 (*‘There are enough people I feel close to’*).

A fourth cluster consisted of nearly all of the UCLA nodes. However, this cluster also contained the SELSA-4 family loneliness item (*‘There is no one in my family I can depend on for support and encouragement, but I wish there was’*), all five of the SELSA-S social loneliness nodes, the direct measure of loneliness, all of the dJG social loneliness nodes and all of the dJG emotional loneliness nodes.

A fifth cluster consisted of three items which were assigned to two clusters each. These were SELSA-1 (third and fifth clusters) (*‘I feel alone when I am with my family’*), UCLA-3 (fourth fifth clusters) (*‘How often do you feel there is no one you can turn to?’*) and dJG-10 (fourth and fifth clusters) (*‘I often feel rejected’*).

Finally, the dJG-1 item (*‘I experience a general sense of emptiness’*) is assigned to three different clusters (second, third and fourth clusters).

### Bridge expected influence estimation

**Fig. 3:**
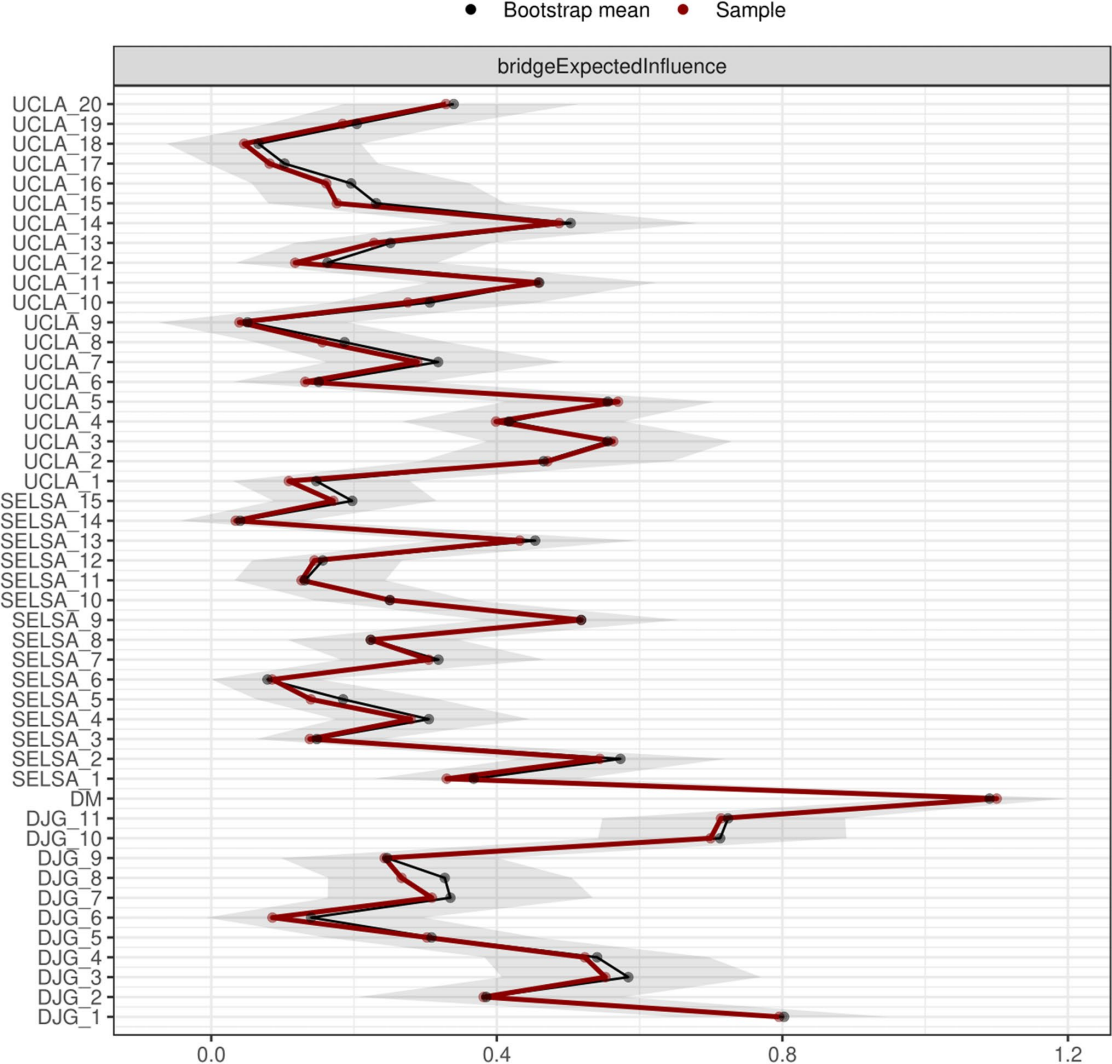
Bootstrapped bridge expected influence estimates for all items

When examining bridge centrality, unsurprisingly the single item direct measure of loneliness had the strongest connectivity to all of the measures, as it is a single item and hence its own cluster. The three nodes that exhibited the next strongest bridge expected influence between the explicitly theoretically defined scale clusters within the network in descending order are as follows: dJG-1 (*’There is always someone I can talk to about my day-today problems’*), dJG-11 (*’I can call on my friends whenever I need them’*) and dJG-10 (*‘I often feel rejected’*). This suggests that these three nodes have the strongest connectivity with other loneliness measures. Following bootstrapping, the dJG-1 item was shown to have stronger bridge expected influence than the dJG-10 item. Further, the bridge expected influence estimate was found to be stable as, following a case resampling bootstrap, the centrality stability coefficient passed the accepted cut off of 0.5 (*CS*(cor = 0.7) = 0.595). See Fig. 3 for bridge centrality estimates.

## Discussion

The present study aimed to, within a sample of older adults, explore the interrelationships between all items from the UCLA-3, SELSA-S, dJG and a direct measure of loneliness via the estimation of a regularised partial correlation network. Further aims were to determine factorial structure within this network and to determine the items with the strongest connectivity between the separate measures of loneliness.

It should be noted that the factor structure recovered for each individual scale is influenced by every scale included in the network. Different clusters (factors) may be found when performing network analysis separately for each scale. These separations may not be distinct enough to be seen when all scales are examined simultaneously, and more overarching clusters may be observed. However, our aim was to determine *shared* clusters (factors) between the scales and to determine similarities and differences in the types of loneliness that each scale is able to assess.

### SELSA—S

Regularised partial correlation network estimation demonstrated that the SELSA-S appears to be distinct from the other measures of loneliness. The romantic loneliness scale items were grouped together with a further distinction between the positively and negatively worded items of the SELSA-S. Clique percolation confirmed this finding by allocating these nodes to separate clusters. This is in contrast to previous work which has found no such separation (Adamczyk & DiTommaso, 2014; DiTommaso et al., 2004). The SELSA-S family loneliness scale was also found to be a distinct entity in both estimations as is indicated in previous work (Adamczyk & DiTommaso, 2014; DiTommaso et al., 2004). The social loneliness subscale of the SELSA-S was also identified as a separate cluster. These findings add some credence to the previously identified three-factor structure of the SELSA-S, albeit with potentially an additional method factor for the romantic subscale. All of the above suggests the utility of the SELSA-S for researchers and clinicians in determining the type of relational deficits that are resulting in the experience of loneliness.

### dJG

For the dJG scale, a separation was observed. A dyad emerged containing the only two nodes which related to rejection and emptiness (dJG-10 & dJG-11). Notably, the two items in this cluster refer only to specific feelings or states with no explicit reference to personal relationships. The remaining nodes all refer to people, others or friends, which could explain the distinction between these two items and others. However, clique percolation did not identify these two items as a separate cluster assigning all nodes to the same cluster. This is in line with previous demonstrations of the unidimensionality of this scale (Buz & Perez-Arechaederra, 2014; Buz et al., 2014; de Jong-Gierveld & Kamphuis, 1985). No distinction was found between items relating to social and emotional loneliness, which does not support the original theoretical intentions of the scale (de Jong-Gierveld & Kamphuis, 1985).

The clique percolation plot identified that both the emotional and social loneliness subscales were incorporated into one large factor which also included all UCLA items and the SELSA social loneliness subscale, suggesting that these items all tap into a similar construct.

Clique percolation further indicated that the dJG-8 node (*‘There are enough people I feel close to’*) was also part of the SELSA-S family loneliness cluster. This indicates that even though the dJG in general appears to be measuring a construct similar to that of the UCLA scale and the SELSA-S social loneliness scale, this particular item is the exception and may be more aligned to family loneliness. Similarly, the dJG-1 node (*‘There is always someone I can talk to about my day-today problems’*) was part of both SELSA-S romantic loneliness clusters. As these are only single items and as none of the dJG items refer specifically to either romantic or family relationships, their usefulness in capturing these relational aspects of loneliness could be limited.

Our findings suggest that if researchers are using the dJG measure they are likely unable to identify aspects of loneliness relating to specific functional social ties. Deficits in romantic relationships, based on marital status, are known to be strong predictors of loneliness (e.g., Child & Lawton, 2019; Golden et al., 2009; Theeke, 2009), as well as its psychological and physiological health correlates (for a review see Smith & Christakis, 2008). Similarly, although research suggests that social loneliness (e.g., deficits in friendships) is more important in relation to loneliness (e.g., Lee & Ishii-Kuntz, 1987; Shiovitz-Ezra & Leitsch, 2010; Silverstein et al., 1996), family relationships are also important in their own right (e.g., Mullins et al., 1987; Pinquart & Sörensen, 2001; Steed et al., 2007). Therefore, if researchers are interested in these relational aspects of loneliness, the dJG may not be as beneficial as other measures.

### UCLA

The UCLA-3 scale was largely grouped together into one unitary structure. This supports the unidimensionality of the UCLA-3 scale as suggested by Russell and colleagues (Russell, 1982, 1996). Our network analyses did not support method factors based on the wording of the items leading to clustering as has been found in previous studies (Boffo et al., 2012; Durak & Senol-Durak, 2010; Russell, 1996). As mentioned above, the clique percolation network method grouped the UCLA-3 further together with the SELSA-S social loneliness subscale and the dJG social and emotional subscales. No UCLA items were part of the romantic loneliness cluster. This could suggest that, like the dJG, the UCLA scale may not capture romantic loneliness. Further, only the UCLA-10 item (*‘How often do you feel close to people?’*) was part of the SELSA-S family loneliness cluster as well as the larger loneliness cluster. This indicates that this particular node may tap into the family aspect of loneliness. However, as this is only one item and none of the UCLA items explicitly mention family relationships, the usefulness of this node in capturing this family aspect is limited. The same issues mentioned above of a lack of capturing these relational aspects for the dJG thus apply here also.

It is possible that the main loneliness cluster, identified via clique percolation, is solely measuring social loneliness. At first glance, the inclusion of the dJG emotional loneliness subscale might indicate that this cluster is indicative of both social and emotional loneliness. Yet, based on closer inspection of the dJG emotional loneliness items it is clear that none of these items explicitly refer to deficits in specific emotional attachments. Indeed, only one item refers to a close relationship, rather than a deficit in social interactions or feelings; namely emptiness or rejection. Similarly, the items from the UCLA scale only rarely refer to a close relationship and do not further refer to emotional attachment. Based on this and the inclusion of both the UCLA scale and both subscales of the dJG we suggest that the dJG and UCLA measures of loneliness by and large assess social loneliness. It seems, therefore, that should a researcher wish to capture deficits in specific relationships, such as family or romantic attachments, that the SELSA-S should be the preferred measure. The other measures (dJG, UCLA) are suitable for research where measuring social loneliness is key.

The clique percolation estimation identified a further cluster consisting of three nodes that belonged to two groups each. These were SELSA-1 (*‘I feel alone when I am with my family’*), UCLA-3 (*‘How often do you feel that there is no one you can turn to?’) and dJG-1 (‘There is always someone I can talk to about my day-today problems’*).

### Edge weight estimation

In regard to specific connections within the networks, the strongest edge weight in relation to the direct measure of loneliness was found in connection to the UCLA-4 node which states ‘*How often do you feel alone*’. This is perhaps unsurprising given the similarity of wording between the UCLA-4 item and the direct measure of loneliness; which states ‘Do you feel lonely?’ with similar frequency response options. Interestingly, the wording of both the direct measure and the UCLA-4 item are aligned very closely to the single item loneliness measure recommended by the Office for National Statistics (*How often do you feel lonely?*) (Snape & Martin, 2018). Similar to the points regarding the UCLA-3 and dJG scales, the finding that this direct measure links most strongly to items which fall within the large social loneliness cluster and only very weakly, if at all, to any node from the romantic and family subscales of the SELSA-S suggests that the direct measure of loneliness may not tap into a lack of any specific personal relationship, but rather social loneliness. This recommended measure is perhaps not adequate for assessing loneliness in relation to deficits in specific emotional attachments or relationships.

### Bridge expected influence estimation

The dJG-1 item (*‘There is always someone I can talk to about my day-to-day problems’*) demonstrated the strongest bridge expected influence after the direct measure. This suggests that this particular node has the strongest connectivity to the other measures of loneliness (See Fig. 3). Clique percolation further supported this finding, showing that this node was part of three different clusters (See Fig. 2). It belonged to the large social loneliness cluster as well as both of the romantic loneliness (positively and negatively worded) clusters. Interestingly, although the direct measure of loneliness demonstrated the most bridge expected influence (See Fig. 3), as mentioned above, clique percolation did not assign it to more than one cluster (See Fig. 2). This further suggests that this item does not tap into the family and romantic relationship aspects of loneliness. Perhaps the dJG-1 item could thus also be suited as a direct measure of loneliness as our findings suggest that this item taps into both romantic and social loneliness. However, as with the currently used direct measure this item does not seem to tap into family based loneliness.

The dJG-11 (*‘I can call on my friends whenever I need them’*) and the dJG-10 (*‘I often feel rejected’*) items also demonstrated comparatively stronger bridge expected influence than others suggesting that these items have strong connectivity to the other measures. However, these findings were not further corroborated by the clique percolation plot.

### Implications

As highlighted, our findings point to the utility of the SELSA-S in assessing loneliness based on specific relational deficits. This means that should researchers and clinicians be interested in identifying the specific relational, or to assess the success of an intervention targeting specific relationships, then the SELSA-S is likely to be the most useful instrument of choice.

Importantly, it does not appear that the UCLA or dJG scales are able to tap into loneliness resulting from deficits in these specific attachments. This is despite the latter being modelled on Weiss’ typology of social and emotional loneliness. This has implications for scale choice as choosing the dJG scale may not fully capture what the researcher or practitioner is intending to assess.

### Strengths and limitations

The present study employed a novel approach to identify the associations within and between the commonly used measures of loneliness. In doing so, it has been highlighted that if particular relationships are important for a research question, then the SELSA-S may be able to provide answers that the other measures cannot. The study has also indicated that a commonly used direct measure of loneliness aligns most strongly to the UCLA-4 item and has suggested that the dJG-1 node may be a beneficial alternative as it has the strongest connectivity between measures suggesting it may tap into loneliness resulting from deficits in a greater variety of relationships. It is also important to note that for our analyses, we included multiple measures as we were primarily interested in associations between different scales and their items. The structure found could be different if analyses are conducted for each scale separately.

Despite these novel findings and implications, the study is not without limitations. Although a sample of older adults was recruited from across the United Kingdom. We limited our sample to those individuals without clinical diagnoses for anxiety and depression. It is unclear whether our findings would generalize to clinical samples. Recruitment was also limited to those with access to the internet. Many older adults do not have this access (Yu et al., 2016) and/or autonomy with internet use (Hargittai et al., 2019) and so the current findings are not wholly representative of the UK population. However, as noted by Peer and colleagues (2017), the use of Prolific as a crowd sourcing participant recruitment platform may result in a more diverse sample of participants in comparison to other platforms such as MTurk. Despite this diverse sample, it is possible that only including those with internet access may have resulted in higher levels of loneliness being reported on average across the scales. This could be due to the previously established link between internet usage and lower reported levels of both social and emotional loneliness (e.g., Cotten et al., 2013; Sum et al., 2008).

Further, the present study has also not explored whether the structure of this network of loneliness measures is equivalent across gender, age or other factors, such as educational attainment. This equivalence is important should researchers wish to use such measures to draw comparisons about loneliness across these domains. Therefore, it would be beneficial for the study to be repeated incorporating a representative sample of older adults within the UK and to also test the invariance of the network across age, gender, and other relevant characteristics. Finally, the data presented here were collected prior to the start of the COVID-19 pandemic and therefore before any lockdown measures and isolation procedures were implemented in the UK. This reduction in contact with one’s social network may have induced perceptions of deficits in emotional and social attachments resulting in higher levels of loneliness. The ability to determine which type of attachment deficit loneliness has resulted from via a particular scale would be beneficial here. This would allow appropriate interventions to be employed to bolster these attachments.

In summary, the present study aimed to determine which aspects of loneliness each loneliness scale was able to assess as well where the scales overlap in order to facilitate research and practitioner scale choice. Network analysis indicated that the SELSA-S may be the only of the included measures with the ability to tap into loneliness resulting from deficits in particular relationships. Should researchers be interested in investigating loneliness associated with specific relationships, it would be beneficial for them to use the SELSA-S as their chosen measure. However, if loneliness more generally is of interest, then the dJG, UCLA or the direct measure of loneliness may be suitable. The study also highlighted the importance of dJG 1 item in having the most connectivity with other measures. Future work can explore whether this single item could be an alternative direct measure of loneliness than those currently utilised.

## Data Availability

Data is available at https://osf.io/5f2ph/?view_only=fb99e7e8c41f427faa86b7167dcf1fe9.
